# Mechanisms of 5-HT receptor antagonists in the regulation of fibrosis in a 3D human liver spheroid model

**DOI:** 10.1038/s41598-023-49240-9

**Published:** 2024-01-16

**Authors:** Sara Redenšek Trampuž, Sander van Riet, Åsa Nordling, Magnus Ingelman-Sundberg

**Affiliations:** 1 Section of Pharmacogenetics, Department of Physiology and Pharmacology, Karolinska Institutet, 17177 Stockholm, Sweden; 2 Pharmacogenetics Laboratory, Institute of Biochemistry and Molecular Genetics, Faculty of Medicine, University of Ljubljana, Vrazov trg 2, 1000 Ljubljana, Slovenia

**Keywords:** Drug discovery, Gastroenterology

## Abstract

Non-alcoholic steatohepatitis (NASH) is a major health problem leading to liver fibrosis and hepatocellular carcinoma, among other diseases, and for which there is still no approved drug treatment. Previous studies in animal models and in LX-2 cells have indicated a role for serotonin (5-HT) and 5-HT receptors in stellate cell activation and the development of NASH. In the current study, we investigated the extent to which these findings are applicable to a human NASH in vitro model consisting of human liver spheroids containing hepatocytes and non-parenchymal cells. Treatment of the spheroids with 5-HT or free fatty acids (FFA) induced fibrosis, whereas treatment of the spheroids with the 5-HT receptor antagonists ketanserin, pimavanserin, sarpogrelate, and SB269970 inhibited FFA-induced fibrosis via a reduction in stellate cell activation as determined by the expression of vimentin, TGF-β1 and COL1A1 production. siRNA-based silencing of 5-HT_2A_ receptor expression reduced the anti-fibrotic properties of ketanserin, suggesting a role for 5-HT receptors in general and 5-HT_2A_ receptors in particular in the FFA-mediated increase in fibrosis in the human liver spheroid model. The results suggest a contribution of the 5-HT receptors in the development of FFA-induced human liver fibrosis with implications for further efforts in drug development.

## Introduction

Non-alcoholic fatty liver disease (NAFLD) is the most common liver disease worldwide, affecting between 20 and 30% of the population, with the highest prevalence in Western countries^1^. NAFLD presents as a continuum of different conditions, and approximately 25% of patients go on to develop non-alcoholic steatohepatitis (NASH), liver fibrosis, cirrhosis or hepatocellular carcinoma^2–6^. NASH is characterized by a further increase in steatosis, inflammation, hepatocellular ballooning, and increased deposition of extracellular matrix (ECM). The deposited ECM is produced in large quantities by activated hepatic stellate cells (HSCs). The excessive deposition of ECM forms fibrotic scars that disrupt the architecture of the liver, thereby impairing its function^7^.

Serotonin (5-hydroxytryptamine; 5-HT) is a monoamine neurotransmitter with important functions in various physiological processes. It binds to the 5-HT receptor family which is expressed in various subtypes throughout the body; 5-HT_2A_ and 5-HT_2B_ receptors are predominantly expressed in the liver^8^. Ninety percent of endogenous 5-HT is produced in the enterochromaffin cells in the intestinal epithelium^9,10^. In human plasma, most of 5-HT is stored in and released from platelets, where its concentration is up to 10 μM^11^. In addition, serum concentration of 5-HT can reach 4 μM, which increases somewhat in NAFLD patients^12^, and furthermore 5-HT concentration in the portal vein can reach up to 1.2 μM^13^. However, there is a gap in our knowledge regarding the signalling and function of 5-HT in the liver, particularly with regard to its role in fibrosis^13^.

Regarding the possible effect of 5-HT in hepatic steatosis and fibrosis, selective 5-HT_2_ receptor antagonists have been described to induce apoptosis of activated HSCs in rats and to suppress proliferation of HSCs after partial hepatectomy^14^. In experiments with LX-2 cells, the 5-HT_2A_ receptor antagonists ketanserin and sarpogrelate reduced viability and wound healing and also decreased the expression of α-smooth muscle actin (α-SMA) and pro-collagen type I^15^. In addition, ketanserin reduced transforming growth factor-beta 1 (TGF-β1) and pro-collagen type I N propeptide (PINP) in a mouse model of liver fibrosis induced by CCl_4_^16^. In addition, gut-derived metabolites of 5-HT have been suggested to be associated with the risk of developing NAFLD, and studies in mice suggest a role for the 5-HT receptors in the development of steatosis^13,17^. Evidence in rats suggests that gut-derived 5-HT contributes to the progression of NASH via the 5-HT_2A_/PPARγ2 pathway in the liver, where e.g. 5-HT receptor antagonists reduced NAFLD progression^12^, while its effect in human liver is still unknown. Indeed, interspecies differences in the development and progression of liver fibrosis are pronounced and this difference contributes to the fact that no drug for NASH is yet on market.

We have previously described a 3D spheroid model that mimics the human liver in vivo in terms of proteomic, transcriptomic, and metabolomic data^18,19^. Based on this model we have developed a human liver spheroid NASH model that mimics free fatty acid (FFA) induced liver fibrosis. In previous studies, we have demonstrated that this human 3D spheroid model when treated with FFA becomes steatotic and develops a fibrotic phenotype with an increased COL1A1 deposition, TGF-β1, CTGF, and CYP2E1 production. We showed that the model can be used to study pathogenesis of NASH and liver fibrosis and for drug screening to treat NASH^20,21^. In this study, we used the model to investigate the role of 5-HT, 5-HT signalling pathways, and the 5-HT_2A_ receptor in the development of FFA-induced liver fibrosis. The data suggests that 5-HT and the 5-HT_2A_ receptor contribute to increased collagen expression and that 5-HT receptor antagonists reduce liver fibrosis in this model.

## Methods

### Ethical aspects

All cells used in the present study were purchased from the companies listed below. Their documentation below indicates that all cells used in the present study are obtained on the basis of informed consent from all subjects who provided their livers. (1) BIOIVT Certificate Donor consent, 21 May 2018 (BioIVT), (2) BIOIVT Ethics Policy Statement, April 2018, (3) KaLy Cell CONSENTEMENT GENERAL DU PATIENT 16/10/08, (4) Attestation and Redacted OPO consent form (KaLy Cell) and (5) Lonza human tissue letter, Sept 2017.

### Spheroid cultures

Cryopreserved primary human hepatocytes (PHH) and crude non-parenchymal cells (NPC) were obtained from KaLy-Cell (KLC; Plobsheim, France) and Lonza (Basel, Switzerland). NPC were passaged as previously described^22^. Donor characteristics of PHH and NPC are listed in Table 1. PHH were seeded in 96-well Corning® Costar® Ultra-Low Attachment Plates (Merck, Kenilworth, NY) or 96-well Nunclon™ Sphera™ U-Shaped-Bottom Microplate (Thermo Fisher Scientific, Waltham, MA) with NPC at a cell ratio of 4:1 (1500:375) as previously described^18,22^. Spheroids were cultured in William's E medium (Thermo Fisher Scientific) supplemented with 2 mM L-glutamine (Sigma-Aldrich, Saint Louis, MO), 100 units/mL penicillin (Sigma-Aldrich), 100 μg/mL streptomycin (Sigma-Aldrich), 100 nM dexamethasone (Sigma-Aldrich), ITS X-100 (Thermo Fisher Scientific) and 10% fetal bovine serum (FBS; Thermo Fisher Scientific). The medium was changed 5 days after seeding and every 2–3 days thereafter using the medium described above without adding FBS. Induction of liver fibrosis and treatment of spheroids with various substances was initiated 7 days after seeding. The spheroids were harvested on day 14. Spheroids were cultured in 100 μl medium under standard cell culture conditions at 37 °C in a humidified incubator at 5% CO_2_. Unless otherwise stated, the combination of PHH from donor 1 and NPC from donor 6 was used.

**Table 1:** Characteristics of primary human hepatocyte and non-parenchymal cell donors.

Donor	PHH	NPC	Origin	Cat. No	Age	Sex	Ethnicity	*PNPLA3* rs738409	*PNPLA3* rs2294918
Donor 1	X		KLC	S1506T	47	Female	Caucasian	Het	WT
Donor 2	X		Lonza	HUM183061	54	Male	Caucasian	Het	Hom
Donor 3	X		Lonza	HUM183351	56	Female	Asian	Het	Het
Donor 4	X		Lonza	HUM190171	48	Female	Caucasian	Het	Hom
Donor 5	X		Lonza	HUM201221	51	Female	African American	WT	Het
Donor 6		X	KLC	S1493	63	Male	Caucasian	WT	WT
Donor 7		X	Lonza	HUCNP#182,001	55	Female	Caucasian	WT	WT

*WT* homozygous for the reference allele of the respective polymorphism, *Het.* heterozygous for the respective polymorphism, *Hom.* homozygous for the alternative allele of the respective polymorphism.

### Induction of a NASH-like phenotype

Spheroid cultures were exposed to a lipogenic cocktail of FFA, as previously described^23^, with minor modifications. Unsaturated oleic acid (Sigma-Aldrich) and saturated palmitic acid (Sigma-Aldrich), solubilized in ethanol, were conjugated to 10% bovine serum albumin (BSA; Sigma-Aldrich) at a 1:5 molar ratio for 2 h at 40 °C. The FFA were combined in a 1:1 ratio. Spheroids were treated with 480 µM FFA from day 7 to day 14, with medium changed every 2–3 days.

### Functional responses

**Figure 1:**
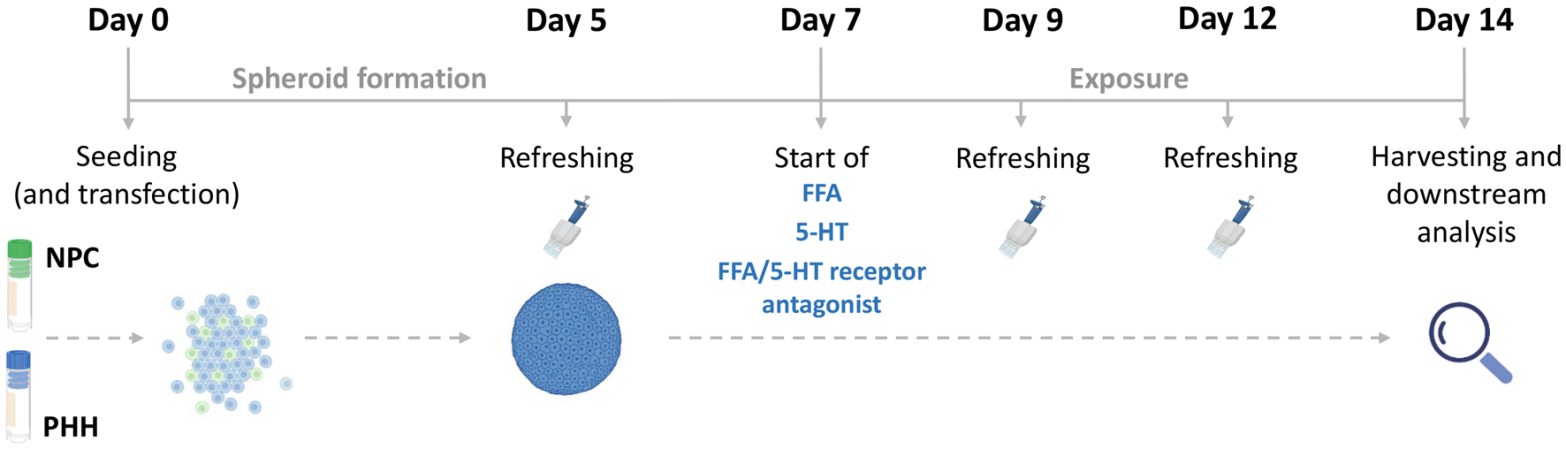
Schematic overview of experimental setup. The cells were seeded on day 0 and the first medium refreshment took place on day 5. All treatments of FFA, 5-HT, FFA/5-HT receptor antagonist, started on day 7 and lasted for 7 days. Medium and compounds were refreshed every 2–3 days, until the spheroids were harvested. In experiments, where cells were transfected, this took place on day 0 along with the seeding procedure. *NPC* non-parenchymal cells, *PHH* primary human hepatocytes, *FFA* free fatty acids, *5-HT* serotonin. Parts of the figure were created with BioRender.com.

Spheroids were treated with several different compounds, all dissolved in dimethylsulfoxide (DMSO): 10 μM serotonin hydrochloride (H9523; Sigma-Aldrich), 1.25 μM ketanserin tartrate (S006; Sigma-Aldrich), 5 μM sarpogrelate hydrochloride (3739; Tocris Bioscience), 1 μM pimavanserin (HY-14557; MedChemExpress, Monmouth Junction, NJ), and 10 μM SB-269970 hydrochloride (1612; Tocris Bioscience). The final DMSO concentration in the medium was 0.1%. A general experiment layout is presented in the Fig. 1.

### Culturing primary human non-parenchymal cells in 2D

NPC were thawed and seeded in 8-well slide chambers (Thermo Fisher Scientific) using the same medium as described for spheroids. After 48 h, the cells were fixed. Cells were cultured in 300 μL medium under standard cell culture conditions at 37 °C in a humidified incubator at 5% CO_2_.

### Cell viability

ATP content was measured on the day of harvest in at least 4 spheroids per condition as previously described^21^. The CellTiter Glo Luminescent Cell Viability Assay Kit (Promega, Madison, WI) was used according to the manufacturer’s instructions. First, 80 μL of medium was removed from the wells and then 25 μL of reconstituted assay reagent was added. The spheroids were mechanically disrupted by pipetting and the plate was incubated at 37 °C for 20 min in the dark. The luminescence signal was measured using a MicroBeta LumiJET 2460 Microplate Counter (Perkin Elmer, Waltham, MA) in white 96-well flat-bottomed plates.

### RNA isolation and cDNA synthesis

Total RNA isolation was performed with QIAzol lysis reagent (Qiagen, Hilden, Germany) using 48 spheroids essentially as previously described^21^. After addition of the lysis chloroform, the mixture was thoroughly mixed and centrifuged. The aqueous phase was mixed with an equal volume of isopropanol. After centrifugation, the supernatant was discarded and the pellet was washed twice with 70% ethanol. The dried pellet was resuspended in ddH_2_O. RNA concentration was determined using the Qubit 4 fluorometer (Thermo Fisher Scientific). RNA was reverse-transcribed into cDNA with SuperScript III reverse transcriptase (Thermo Fisher Scientific) using a SimpliAmp thermal cycler (Thermo Fisher Scientific) according to the manufacturer's protocol. Up to 500 ng of total RNA per sample was used for cDNA synthesis.

### Gene expression analysis

Amplification reactions were performed using a 2 × TaqMan Universal PCR mix (Thermo Fisher Scientific) on a 7500 Fast Real-Time PCR system (Applied Biosystems, Waltham, MA) with 20 × TaqMan probes (Supplementay Table 1). Gene expression was analyzed using the delta-delta Ct method (2^−ΔΔCt^) with genes of interest normalized to *TBP*.

### Immunohistochemistry

The immunohistochemistry was performed essentially as previously described^21^. Spheroids were fixed for 2 h at room temperature in 4% paraformaldehyde. The spheroids were cryoprotected with 30% sucrose for at least 2 days at 4 °C and subsequently embedded and frozen in the Tissue-Tek OCT (Sakura Finetek, Alphen aan den Rijn, The Netherlands) compound and sectioned at 8 μm. Slides with spheroid sections were blocked with 5% BSA, 0.25% Triton X-100 in PBS (PBS/BSA/Triton; Sigma-Aldrich) for 2 h at room temperature. The list of antibodies can be found in Supplementary Table 2.

The antibodies were diluted in PBS/BSA/Triton. Staining with primary antibodies was performed overnight at 4 °C. Subsequently, the slides with the spheroids were washed 3-times with PBS. The secondary antibody was diluted in PBS/BSA/Triton and staining was performed for 2 h at room temperature in the dark. Slides were mounted using ProLong Gold Antifade Mountant with DAPI (Thermo Fisher Scientific) and then imaged using Olympus IX73 inverted microscope (Olympus, Tokyo, Japan) and processed using StreamView software version 1.9.4 and ImageJ software. A total of 10 to 20 sections of different spheroids were imaged per condition and experiment. For quantification, the integrated density of the corresponding channel was divided by the area of DAPI staining, which indirectly indicates the number of cells. The latter is referred to as fluorescence intensity in the diagrams. The size of the spheroids is marked with a white scale bar of 100 μm.

### Immunohistochemistry staining of 2D cultured non-parenchymal cells

After 2 days, cells were washed with PBS, and fixed in 4% paraformaldehyde for 15 min at room temperature. Immunohistochemistry (IHC) staining was performed as described above, up until the mounting step. Nuclei were stained with Hoechst 33342 (Thermo Fisher Scientific), diluted in PBS at 1:1000, for 10 min. After that, wells were washed with PBS 3-times. Cells were imaged using the Olympus IX73 inverted microscope. Images were processed using StreamView software version 1.9.4 and ImageJ software. The scale bar in the images corresponds to 50 μm.

### Quantification of 5-HT in the lysates and supernatant samples by ultra high-performance liquid chromatography tandem mass spectrometry (UHPLC-MS/MS)

Concentrations of 5-HT in the samples were measured by UHPLC-MS/MS following derivatization with benzoyl chloride as described elsewhere^24,25^. Briefly, 10 µl volumes of the deproteinated supernatants were derivatized with 10 µl benzoyl chloride (2% in ACN) in the presence of 0.1 M sodium carbonate (pH 10.5) at room temperature for 3 min. The reaction was terminated by pipetting 20 µl ammonium formate (0.3 M; pH 4.1) and 10 µl DMSO:H_2_O (1:1), 10 µl of the final solution was injected on column. The UHPLC-MS/MS system included a Waters Xevo TQ-S micro triple quadrupole mass spectrometer with the electrospray ionization source operating in a positive mode, and an ACQUITY UPLC system (all purchased from Waters Corporation, Milford, MA, USA). The calibration curve was constructed in the range of 0.1–10.2 nmol/l, the limit of quantification of 5-HT in the lysates and supernatants was 0.6 nmol/l.

### Statistical analysis

Quantitative data were analyzed using GraphPad Prism version 9 (GraphPad Software, San Diego, CA) and described as the mean and standard error of the mean (SEM). Statistical analysis of differences induced by stimuli was carried out using a paired, two-sided *t*-test and comparing each condition to control.

## Results

### Free fatty acid-induced fibrogenic response in 3D primary human liver spheroids

**Figure 2:**
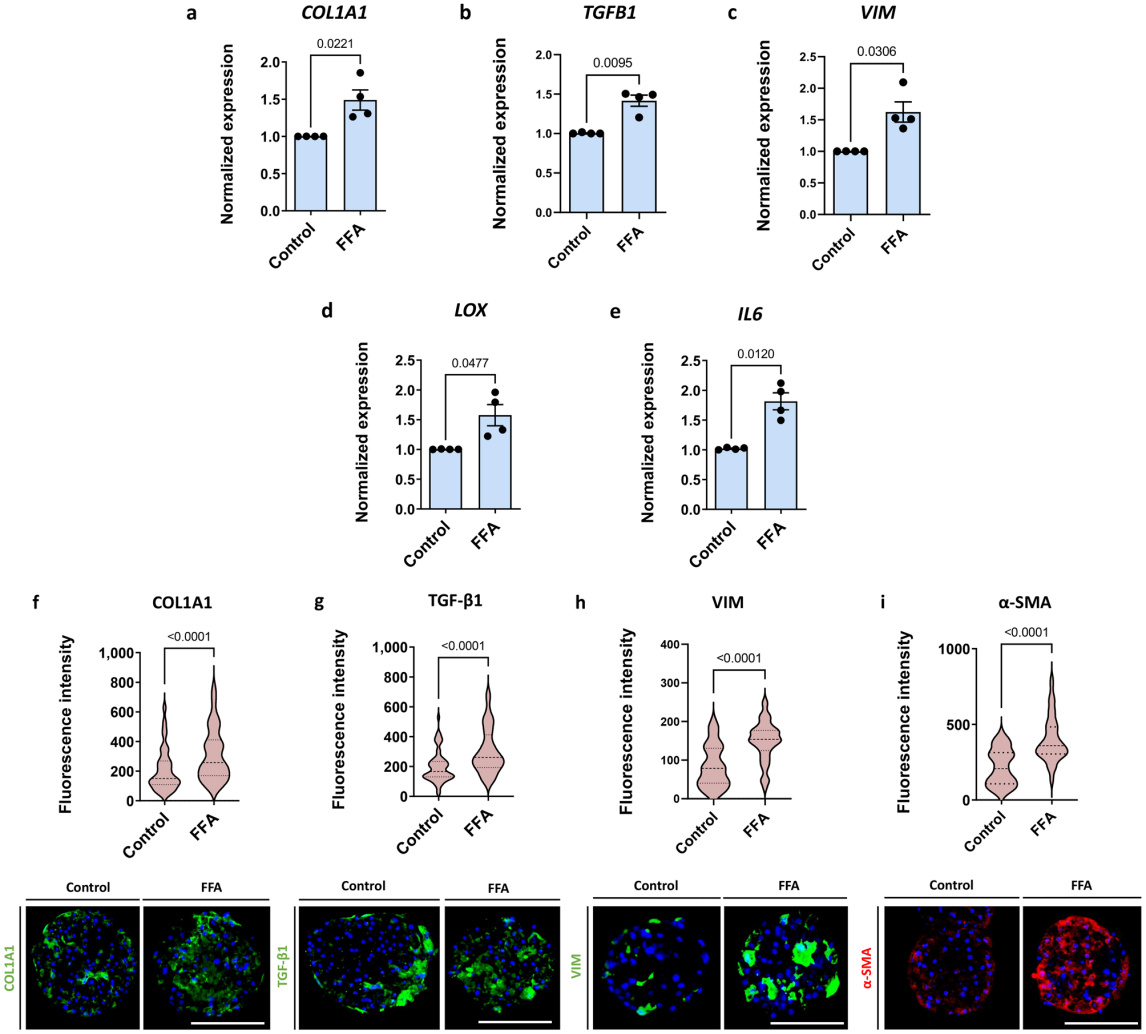
3D human liver spheroids comprised of hepatocytes and non-parenchymal liver cells can mimic the pathology of hepatic fibrosis. To assess the propensity of the system to develop liver fibrosis-like features, the spheroids were treated with FFA for 7 days. (**a–e**) Fibrosis-related genes *COL1A1, TGFB1*, *VIM, LOX*, and *IL6* were upregulated upon FFA treatment (n = 4). (**f–i**) Representative images of COL1A1, TGF-β1, vimentin, and α-SMA deposition in the control or FFA-treated spheroids show an increase following a 7-day treatment with FFA (n = 4). Blue bars represent mRNA expression data. Data is shown as mean ± SEM. Red violin plots show results of IHC analysis, where plots are created based on the fluorescence intensity (integrated density of the corresponding channel divided by the area of DAPI staining) of 10–20 sections of different spheroids per experiment. The number of experiments included in the plot is defined by n. Nuclei in the IHC images are shown in blue. The size of the spheroids is marked with a white scale bar of 100 μm. Data is shown as median with interquartile range. The width of each curve corresponds with the approximate frequency of data points in each region. All experiments were conducted with Donor 1 as the PHH donor and with two different NPC donors, either Donor 6 or Donor 7. Differences were tested using the student's *t* test.

Fibrosis is induced in our liver spheroid fibrosis model containing hepatocytes and NPC by treatment with FFA, for 7 days starting on day 7 after seeding (Fig. 1). In the system presented, treatment with FFA had no effect on ATP or albumin levels, indicating no loss of viability or functionality (Supplementary Fig. S1). Treatment with FFA resulted in increased expression of fibrosis-associated genes such as collagen type I, alpha 1 (*COL1A1*), transforming growth factor-beta 1 (*TGFB1*), vimentin (*VIM*), lysyl oxidase (*LOX*), and interleukin 6 (*IL6*) (Fig. 2a–e). An increase in expression was found at the protein level, for COL1A1, TGF-β1, and vimentin as well as for α-SMA (Fig. 2f–2i). The response of the individual spheroids was highly variable, and 10–20 different spheroids were analysed per condition per experiment (Supplementary Fig. S2).

### Serotonin induces a fibrosis-like phenotype in 3D primary human liver spheroids

**Figure 3:**
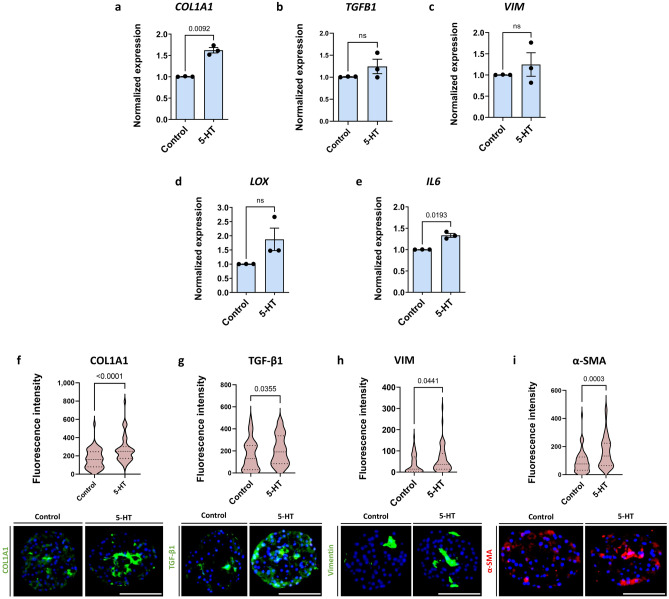
5-HT treatment results in an increase of some fibrotic markers. The spheroids were treated with 10 μM 5-HT for 7 days. (**a, f**) COL1A1 was significantly increased at both mRNA and protein levels (n = 3). (**b, g**) TGF-β1 was consistently increased across experiments due to 5-HT as well. However, the increase was not significant in the case of the mRNA data. (n = 3). (**c, h**) Vimentin was increased following 5-HT treatment on the protein level, but not at the mRNA level. (n = 3). (**d**) *LOX* expression was repeatedly increased upon 5-HT treatment, although not significantly due to high spread (n = 3). (**e**) *IL6* expression was significantly increased on the mRNA level (n = 3). (**i**) α-SMA as an important marker of liver fibrosis was significantly increased upon 5-HT treatment as well (n = 3). Blue bars represent mRNA expression data. Data is shown as mean ± SEM. Red violin plots show results of IHC analysis, where plots are created based on the fluorescence intensity (integrated density of the corresponding channel divided by the area of DAPI staining) of 10–20 sections of different spheroids per experiment. The number of experiments included in the plot is defined by n. Nuclei in the IHC images are shown in blue. The size of the spheroids is marked with a white scale bar of 100 μm. Data is shown as median with interquartile range. The width of each curve corresponds with the approximate frequency of data points in each region. All experiments were conducted with Donor 1 as the PHH donor and with two different NPC donors, either Donor 6 or Donor 7. Differences were tested using the student's *t* test.

The effect of 5-HT on the development of fibrosis was assessed by treating liver spheroids with 10 μM 5-HT according to the scheme in Fig. 1, whereafter several fibrosis-related molecules were quantified. 5-HT treatment did not influence ATP or albumin levels (Supplementary Fig. S3) but increased COL1A1 expression at both mRNA and protein levels (Fig. 3a, f). Furthermore, treatment with 5-HT increased production of TGF-β1, a key regulator of liver fibrosis, slightly but consistently across experiments (Fig. 3b, g). We observed an increase in vimentin, a mesenchymal HSC marker, indicating that 5-HT affects HSC activation status (Fig. 3c, h). Expression of *LOX*, which is important for ECM processing and formation, was increased as well (Fig. 3d). Interestingly, 5-HT treatment also induced the inflammatory component of liver fibrosis as *IL6* expression increased (Fig. 3e). Finally, deposition of α-SMA, a marker of mesenchymal cells, significantly increased upon 5-HT treatment (Fig. 3i). Overall, this suggests that 5-HT is able to increase HSC activation and subsequently ECM deposition independent of FFA. However, the magnitude of this pro-fibrotic effect is smaller in comparison to FFA.

To validate the model as free of endogenous 5-HT, 5-HT was quantified in the lysates and in the supernatant samples, where no 5-HT or amounts very close to detection limit were observed (Supplementary Table 3).

### 5-HT receptor antagonists reduce COL1A1 deposition upon FFA treatment

**Figure 4:**
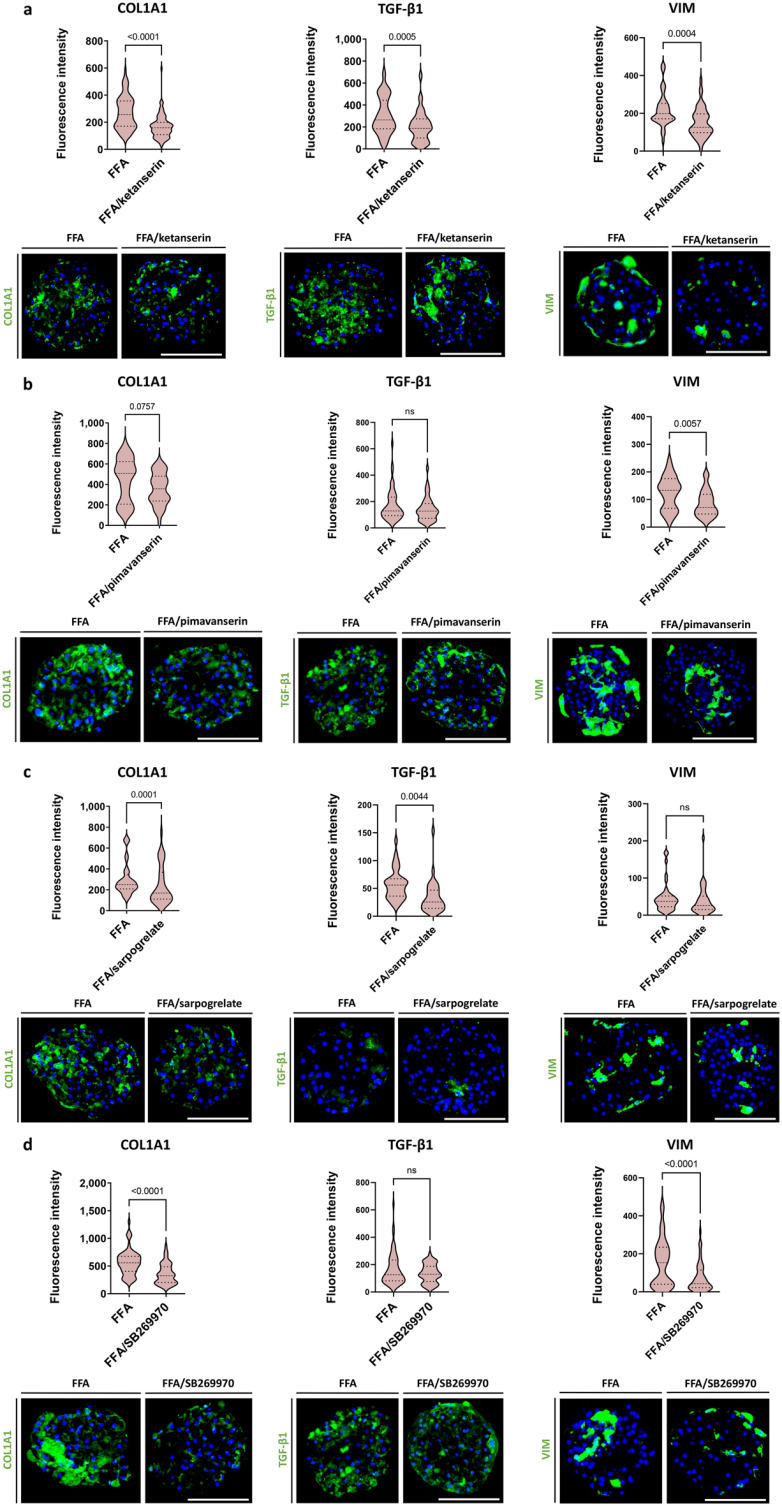
5-HT receptor antagonists can reduce COL1A1 deposition. Different 5-HT receptor antagonists were screened for their anti-fibrotic potential. Ketanserin (5-HT_2A_ and 5-HT_2C_ antagonist), pimavanserin (inverse 5-HT_2A_ agonist), sarpogrelate (5-HT_2A_ and 5-HT_2B_ antagonist), and SB-269970 (5-HT_7_ antagonist) were added to FFA to assess their potential in decreasing COL1A1, TGF-β1, and vimentin production. Representative images of COL1A1, TGF-β1, and vimentin stainings following a 7-day treatment with FFA with or without 5-HT receptor antagonists are presented. (**a**) Ketanserin significantly decreased FFA-induced COL1A1, TGF-β1, and vimentin deposition (n = 4). (**b**) Pimavanserin decreased COL1A1, TGF-β1, and vimentin deposition, although its effect was rather variable and thus non-significant (n = 3). (**c**) Sarpogrelate (n = 3) and (**d**) SB269970 (n = 4; 1 experiment with PHH donor 1, 1 experiment with PHH donor 2, 1 experiment with PHH donor 3, and 1 experiment with PHH donor 4; NPC from donor 6 in all 4 experiments) both significantly attenuated COL1A1 deposition. Sarpogrelate also significantly reduced TGF-β1 production, whereas SB-269970 significantly reduced vimentin. Red violin plots show results of IHC analysis, where plots are created based on the fluorescence intensity (integrated density of the corresponding channel divided by the area of DAPI staining) of 10–20 sections of different spheroids per experiment. The number of experiments included in the plot is defined by n. Nuclei in the IHC images are shown in blue. The size of the spheroids is marked with a white scale bar of 100 μm. Data is shown as median with interquartile range. The width of each curve corresponds with the approximate frequency of data points in each region. Differences were tested using the student's *t* test.

To complement the evaluation of the contribution of 5-HT to fibrosis in hepatic spheroids, we investigated the effect of 4 different 5-HT receptor antagonists, ketanserin, a 5-HT_2A/2C_ receptor antagonist; pimavanserin, an inverse 5-HT_2A/2C_ receptor agonist; sarpogrelate, a 5-HT_2A/2B_ receptor antagonist; and SB269970, a 5-HT_7_ receptor antagonist; generally at 5-times their c_max_ values^15,26–28^ (see Fig. 1 for the experimental design). All antagonists caused inhibition of FFA-induced COL1A1 expression, with ketanserin being the most potent (Fig. 4a–d). Ketanserin was the only antagonist that caused a significant decrease in both TGF-β1 production and vimentin expression. The other three antagonists had similar effects, but the differences observed were not all significant.

### Ketanserin, a 5-HT_2A_ receptor antagonist, presents a potential anti-fibrotic compound

**Figure 5:**
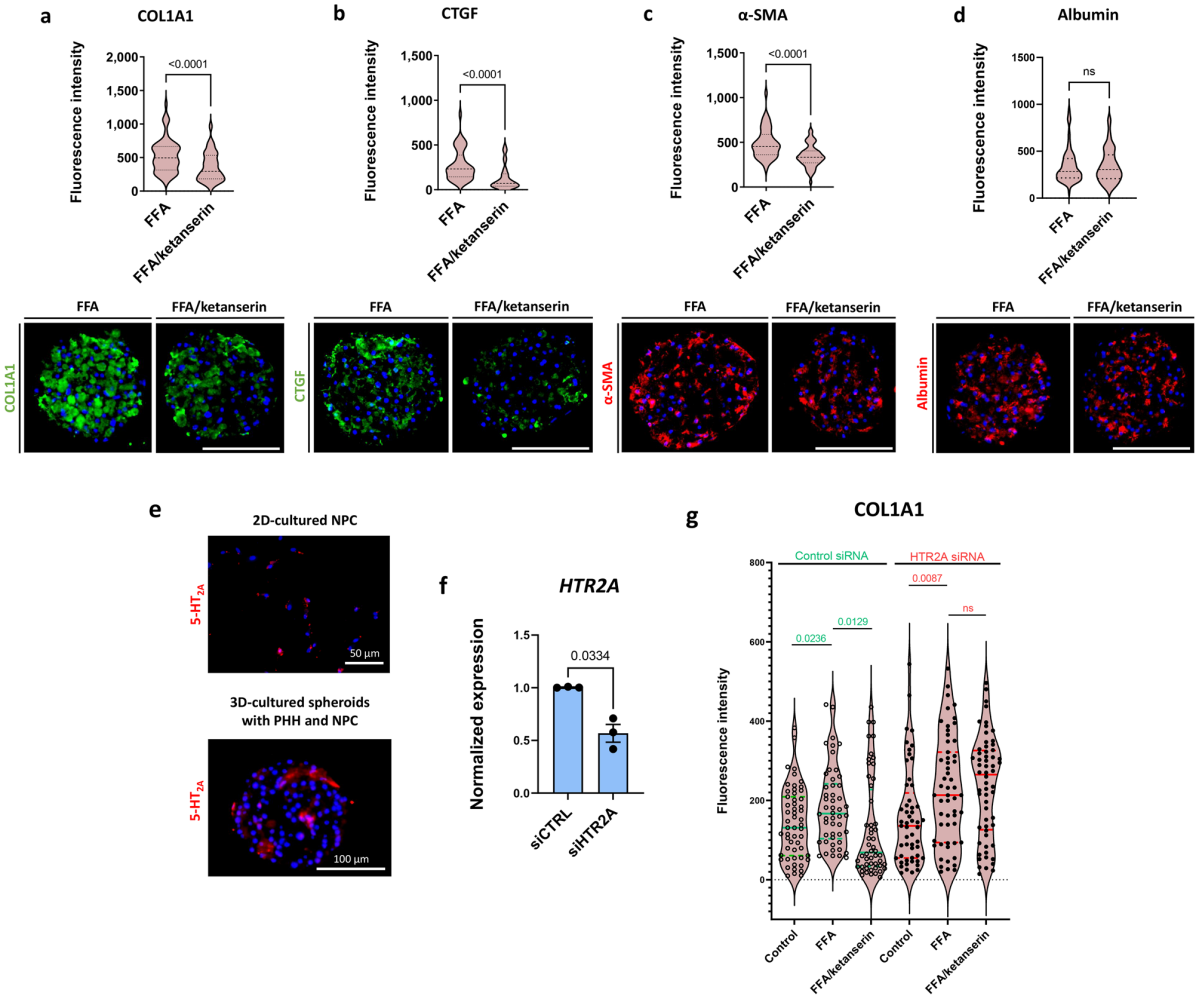
Ketanserin and 5-HT_2A_ signalling inhibition have anti-fibrotic properties. Spheroids were treated with FFA with or without ketanserin from day 7 until day 14. (**a**) Ketanserin reduced the FFA-induced COL1A1 deposition in 4 different PHH donors and 2 different NPC donors (n = 4; PHH donors 1, 2, 3, and 4; NPC donor 6 and 7). (**b**, **c**) Representative images of CTGF and α-SMA show that ketanserin was capable of decreasing these two markers of liver fibrosis (n = 3; PHH donor 1; NPC donor 6 and 7). (**d**) Ketanserin treatment did not affect albumin deposition in the spheroids (n = 3). Violin plots show results of IHC analysis, where plots are rated based on the fluorescence intensity (integrated density of the corresponding channel divided by the area of DAPI staining) of 10–20 sections of different spheroids per experiment. The number of experiments included in the plot is defined by n. Nuclei in the IHC images are shown in blue. Size of spheroids is marked with a white scale bar of 100 μm. (**e**) NPC from Donor 6 were cultured in 2D for 2 days and afterwards stained for 5-HT_2A_. Almost all cells were positive for 5-HT_2A_ (upper panel). Furthermore, 3D spheroids comprised of both PHH (Donor 1) and NCP (Donor 6) were stained for 5-HT_2A_ as well. Only a certain fraction of cells was positive for the receptor (lower panel). The size of the 2D cultured cells is presented with a white scale bar of 50 μm, whereas the size of a spheroid is presented with a white scale bar of 100 μm. Nuclei in the images are shown in blue. (**f**) Afterwards, 5-HT_2A_ was silenced using *5HT2A* siRNA with approximately 45% of reduction in expression. (**g**) Ketanserin significantly reduced the FFA-induced COL1A1 in the control siRNA-treated spheroids, whereas this was not the case in the *5HT2A*-silenced spheroids (n = 3). Every dot in the violin plot represents the quantified fluorescence intensity of one stained section of a spheroid. Data in the violin plots is shown as median with interquartile range. The width of each curve corresponds with the approximate frequency of data points in each region. Differences were tested using the student's *t* test.

As mentioned above, 5-HT is thought to exacerbate liver fibrosis in rodents via signalling through 5-HT receptors expressed by PHH and HSCs, particularly the 5-HT_2A_ receptor^13^. Ketanserin was able to inhibit FFA-induced fibrosis, as indicated by significantly decreased COL1A1, TGF-β1, and vimentin accumulation in hepatic spheroids treated with FFA (Fig. 4a). A significant ketanserin effect with reduced COL1A1 deposition was also observed in spheroids from three other PHH donors (Fig. 5a). Ketanserin also caused a decrease in the production of CTGF, an important mediator of liver fibrosis (Fig. 5b). Finally, ketanserin inhibited the expression of α-SMA (Fig. 5c), indicating a lower degree of epithelial to mesenchymal transition of HSCs within a spheroid. Remarkably, treatment with ketanserin had no effect on the functionality of the hepatocytes, as albumin was not affected by the substance (Fig. 5d).

A crucial aspect is whether the effect of ketanserin and the other 5-HT_2A_ receptor antagonists is mediated by the action of 5-HT_2A_ receptors or by off-target receptors. We found expression of the 5-HT_2A_ receptor by IHC in both 3D spheroids and 2D HSCs (Fig. 5e). Treatment of spheroids with *5HT2A* siRNA at the time of seeding significantly decreased *5HT2A* expression (Fig. 5f). Importantly, siRNA-mediated silencing of the 5-HT_2A_ receptor resulted in inhibition of the ability of ketanserin to reduce FFA-induced COL1A1 expression, suggesting that the 5-HT_2A_ receptor is a likely target for ketanserin action (Fig. 5g).

Taken together, the data based on the decreased ability of ketanserin to reduce FFA-induced liver fibrosis after silencing the 5-HT_2A_ receptor expression suggest, that the 5-HT_2A_ receptor signalling pathways contribute to FFA-induced liver fibrosis in the used human 3D spheroid NASH model.

## Discussion

**Figure 6:**
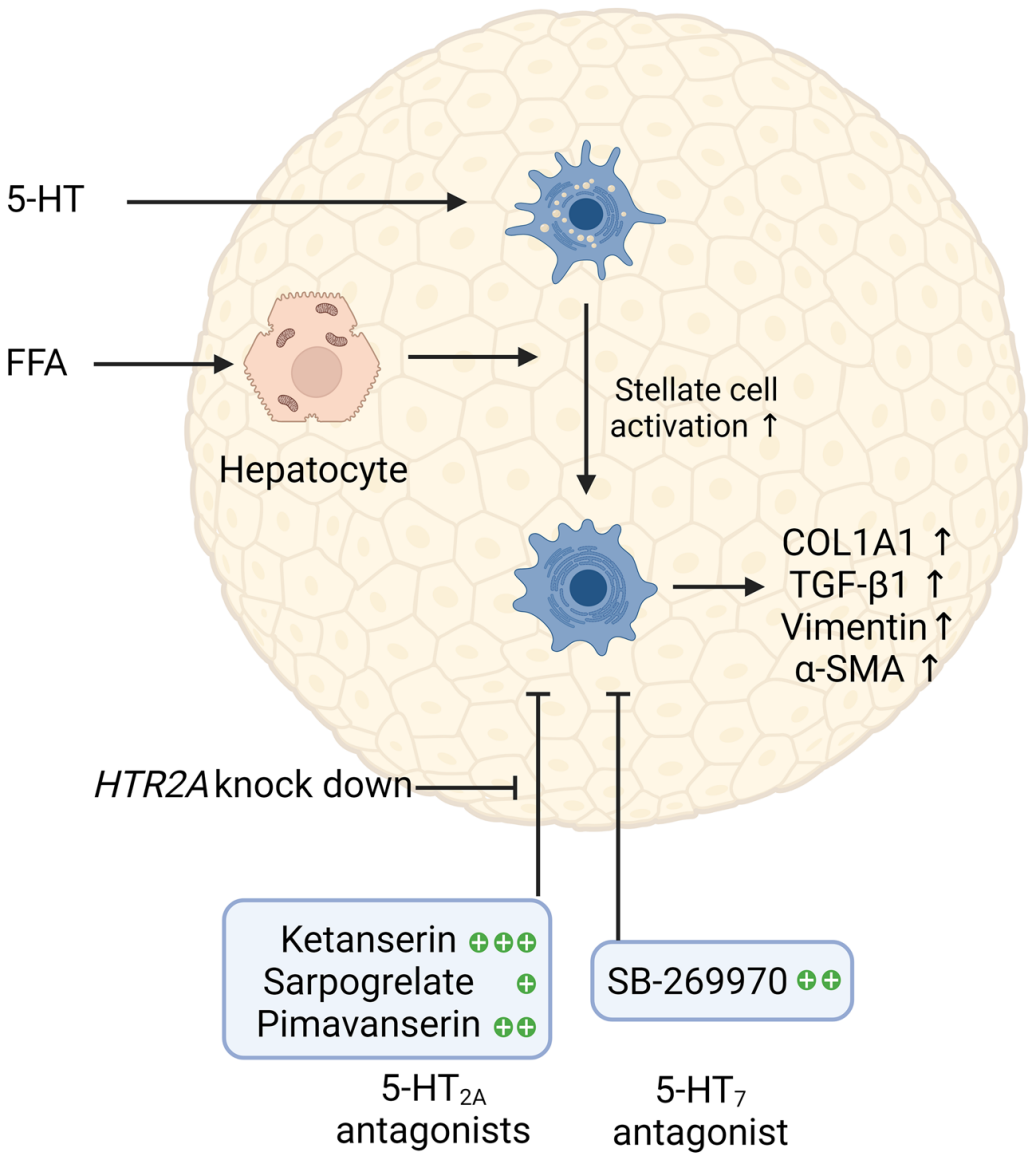
A schematic presentation of the importance of 5-HT signalling in liver fibrosis. Both FFA and 5-HT induced fibrosis in the spheroids, comprised of PHH and NPC, presumably through the activation of hepatic stellate cells. Fibrotic phenotype presented with increased deposition of COL1A1, as well as with increased production of TGF-β1, vimentin, and α-SMA. 5-HT receptor antagonists were tested for their anti-fibrotic capacity. FFA-induced fibrosis was decreased by all four tested 5-HT receptor antagonists. However, ketanserin showed the strongest anti-fibrotic characteristics. Anti-fibrotic activity of the tested 5-HT_2A_ antagonists can be blocked by knocking down the receptor. Results of the study emphasize the importance of 5-HT signalling in liver fibrosis development, as well as warrant further search for novel targets for liver fibrosis treatment among members of the 5-HT pathway. *5-HT* serotonin, *COL1A1* collagen, type I, α1, *FFA* free fatty acids, *NPC* non-parenchymal cells, *PHH* primary human hepatocytes, *TGF-β1* transforming growth factor beta 1. Created with BioRender.com.

We used an in vitro 3D liver spheroid model with PHH and NPC to investigate the role of 5-HT signalling in a model of FFA-induced liver fibrosis, to evaluate the potential of 5-HT receptor antagonists to reduce fibrosis and to modulate the anti-fibrotic effect of antagonists by 5-HT_2A_ receptor siRNA. Our data suggest a pro-fibrotic effect of 5-HT and differential inhibitory effects of four tested 5-HT receptor antagonists (ketanserin, pimavanserin, sarpogrelate, and SB269970) on FFA-induced fibrosis development in the absence of 5-HT. The latter can be reversed by downregulation of the 5-HT_2A_ receptor. The results suggest that it would be useful to search for new drug candidates for the treatment of NASH among the putative targets in the 5-HT_2A_ receptor pathway. A schematic of the overall results is shown in Fig. 6.

The NASH spheroid model used here has been utilized previously for analyses of mechanisms of FFA-induced fibrosis, including the role of CTGF^21^ and the ability of various anti-NASH drug candidates in clinical trials to inhibit the development of fibrosis^20^. The spheroids consisted of hepatocytes and NPC in a ratio of 4:1 which corresponds to the relative composition in human liver. The majority of the crude NPC fraction are HSCs as previously shown by the high proportion of vimentin-positive cells. Furthermore, we have previously shown that there are some liver sinusoidal endothelial cells present in the spheroids at day 14 as some CD32b-positive cells can be detected. However, CD68 can rarely be detected after 14 days in culture, indicating that there are no Kupffer cells present in the spheroid at day 14^20^. The fibrogenic FFA mixture used to induce fibrosis consisted of unsaturated oleic acids and saturated palmitic acids, as these are among the most abundant FFA in human plasma^29^. We used COL1A1 as the primary endpoint, but vimentin, TGF-β1, and α-SMA were also monitored to closely track the state of the HSCs when modelling liver fibrosis.

We found that 5-HT alone is able to significantly induce deposition of COL1A1 and to some extent increase the expression of TGF-β1, vimentin, and α-SMA. Importantly, 5-HT is not produced in the liver, but originates in the gut where it is transported to the liver via platelets, which is supported by the fact that we detected negligible amounts of 5-HT in the culture medium and in the spheroid lysates. Previously, inhibition of 5-HT synthesis in the intestine was shown to ameliorate hepatic steatosis in mice^13,30^, suggesting its role in lipogenesis and lipid accumulation^31^. In addition, rats with a high-fat and high-fructose diet-induced NASH, were treated with 5-HT, whereupon upregulation of lipogenesis-related genes and inflammatory markers was observed^12^. Even though 5-HT levels change in steatotic conditions in liver in vivo, this regulation does not occur in the 3D spheroids since hepatic cells lack the ability of serotonin production. Nevertheless, in the current study, we focused on the role of 5-HT in ECM deposition, which we postulate occurs through activation of HSCs, followed by an increase in COL1A1, vimentin, TGF-β1, and α-SMA expression^6,14^. The mechanisms behind this activation are currently unknown but based on the results of the 5-HT_2A_ receptor knockdown experiment and the effect of the serotonin receptor antagonists in our model, a mechanistic effect related to the action of the 5-HT_2A_ receptor seems likely.

5-HT receptor antagonists targeting the receptors 5-HT_2A_, 5-HT_2B_, and 5-HT_7_ were selected because these receptors are thought to be expressed in the liver^31,32^. Ketanserin and sarpogrelate have been reported to inhibit wound healing in LX-2 cells and induce their apoptosis^15^. In addition, ketanserin showed an anti-fibrotic effect in mice with fibrosis caused by thermal injury^33^. Three possible interrelated anti-fibrotic effects of ketanserin can be deduced from the literature. First, ketanserin interferes with canonical and non-canonical TGF-β1 signalling pathways by decreasing Smad 2/3 phosphorylation^34^ on one hand and JNK and ERK1/2 phosphorylation^35^ on the other, causing a decreased production of ECM proteins. Second, in various animal fibrosis models, ketanserin was shown to decrease the levels of several cytokines such as TNF-α, IL-10, IL-1β, IL-8, and IL-6^33,35,36^. Finally, a study conducted in LX-2 cells showed that ketanserin-induced HSC apoptosis, resulting in decreased α-SMA and pro-collagen I α1 production^15^, both of which aim to alleviate liver fibrosis. Sarpogrelate has been primarily associated with its effects on lipid metabolism^8,13^. However, in a study of CCl_4_-induced acute liver injury in mice, sarpogrelate also affected the phosphorylation of NF-κB, MAPKs (p38, JNK, ERK1/2), and STAT3, as well as the production of the inflammatory factors IL-1β and TNF-α^37^, while its anti-fibrotic properties, as observed in the aforementioned study, have not been previously described. Pimavanserin has never been studied in the context of a chronic liver disease. However, pimavanserin is a more selective agent for 5-HT_2A_ receptor than ketanserin and is an inverse agonist of the receptor^38^. We found that pimavanserin reduced COL1A1 deposition, but to a lesser extent than ketanserin, suggesting that ketanserin exerts some of its anti-fibrotic effects through other mechanisms. Finally, the 5-HT_7_ receptor antagonist SB-269970 showed high efficacy in reducing COL1A1 deposition in all donors. In conclusion, these 5-HT receptor antagonists inhibit the formation of NASH in the spheroid model and further knowledge of their mechanisms of action in relation to the action of the 5-HT_2A_ receptor may be useful in shedding more light on the treatment area of NASH.

Ketanserin showed a consistent anti-fibrotic effect in different PHH donors. The most likely major target of ketanserin is the 5-HT_2A_ receptor. Having demonstrated the presence of this receptor in the model, the anti-fibrotic effect of ketanserin was abolished by downregulating 5-HT_2A_ receptor. This suggests that the 5-HT_2A_ receptor and components of the receptor pathways might be considered as possible anti-fibrotic targets and this item needs further exploration. There are several explanations for the anti-fibrotic properties of ketanserin in the model of fibrosis in human 3D liver spheroids, even without the presence of 5-HT. The 5-HT_2A_ receptor has constitutive activity^39,40^ so its downstream signalling is active even in the absence of its endogenous ligand. Dimerisation interfaces for 5-HT_2A_ receptor oligomers are different when the receptor binds ligands with different pharmacological properties, such as 5-HT as a natural ligand and ketanserin as an antagonist of the receptor^41,42^. In particular, the extent of membrane-directed oligomerisation of a 5-HT_2A_ receptor is predicted to be greater in the ketanserin-bound state than in the 5-HT-bound state^43^. In cardiomyocytes, it has been shown in vitro that heterodimerisation of the 5-HT_2B_ receptor with the β2A receptor is essential for β2A receptor-mediated cardioprotection^44^. Since binding of ketanserin increases the propensity of the 5-HT_2A_ receptor to oligomerise with other receptors, we hypothesise that the 5-HT_2A_ receptor increasingly dimerises with the β2A receptor, as both are G-protein-coupled receptors, which may affect receptor internalisation, cAMP accumulation, and ERK1/2 phosphorylation etc.^43^. All of which contribute to the anti-fibrotic mechanism of ketanserin and presumably other antagonists tested. Another possible factor contributing to the anti-fibrotic effect of ketanserin is the internalisation of the 5-HT_2A_ receptor^45^ triggered by ketanserin. The actual identification of the downstream effects triggered by the 5-HT receptor antagonists requires future experimentation.

This model does come with certain limitations. Although it was feasible to ascertain the relative impact of ketanserin and siRNA constructs on fibrosis progression within the spheroids, accurately gauging the contribution of individual cell types to the accumulation of COL1A1 is challenging due to the structural nature of the spheroids. Consequently, all cell types remain susceptible to the drug- and siRNA-based interventions that were conducted. Moreover, replicating the intricate cell–cell interactions inherent to spheroids, which potentially influence protein and gene regulation, proves intricate with this experimental configuration. Additionally, it is worth noting that, in the majority of instances, PHH donors were specifically chosen for possessing the *PNPLA3* polymorphism I148M. This deliberate selection ensures a robust induction of a fibrotic phenotype but may not be fully representative of the broader human population.

## Conclusions

The results of the present study show that 5-HT receptor antagonists exhibit anti-fibrotic properties, of which ketanserin has the most pronounced effects in the model of FFA-induced fibrosis in primary human 3D liver spheroids. We hypothesise that the effects of the investigated 5-HT receptor antagonists are mediated via the 5-HT_2A_ receptor. This involves receptor dimerisation and downstream signalling pathways that regulate the activation of HSCs, a central aspect of the pro-fibrotic pathway. However, future studies in this area are required to further elucidate the mechanisms of action of 5-HT and 5-HT receptor antagonists in the regulation of liver fibrosis.

### Supplementary Information

Supplementary Information.

## Data Availability

All data generated or analysed during this study are included in this published article (and its Supplementary Information files).
