# Supplementary material for: Mechanisms of 5-HT receptor antagonists in the regulation of fibrosis in a 3D human liver spheroid model
Source: Sci Rep. 2024 Jan 16;14:1396. doi: 10.1038/s41598-023-49240-9 (PMC10792007; doi:10.1038/s41598-023-49240-9)
Supplement: Supplementary file 1 — Supplementary Information. [file 41598_2023_49240_MOESM1_ESM.pdf]

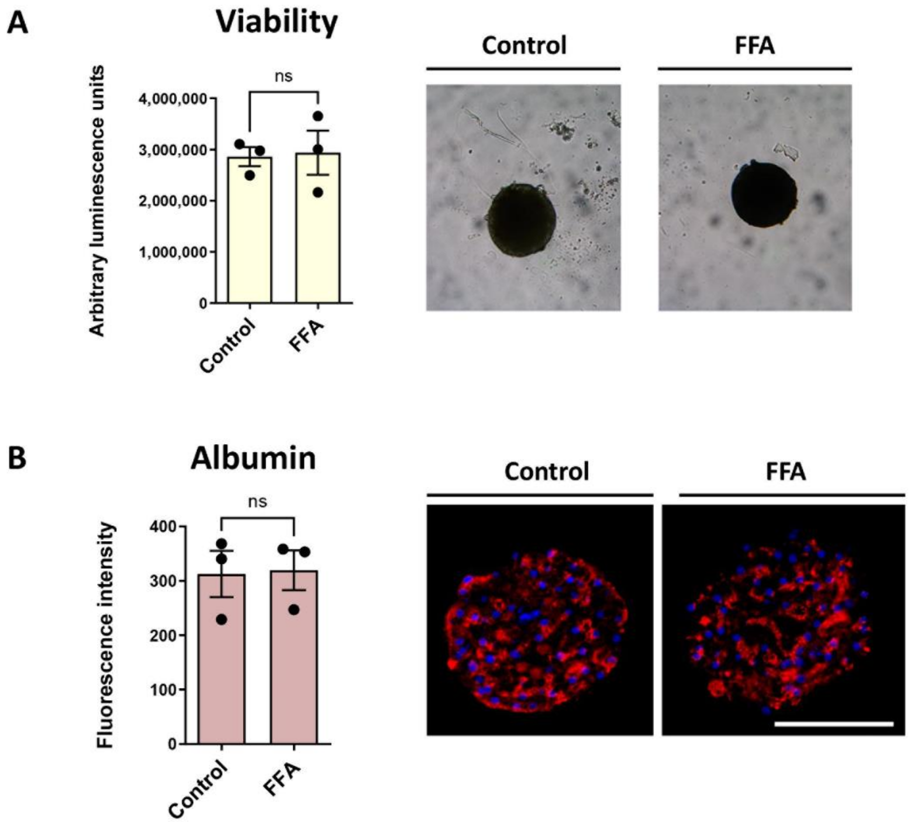

Supplementary Figure S1: Effect of FFA treatment on viability and functionality

(A) Treatment of spheroids with FFA for 7 days did not affect viability of spheroids, which was measured by quantification of the released ATP. One dot represents the average of the ATP measurements in at least 4 spheroids per condition per experiment. (n=3)

(B) Treatment of spheroids with FFA did not affect the functionality of hepatocytes in the spheroids, which was measured by the albumin staining. One dot represents the average of at least 10 imaged spheroids per condition per spheroid. (n=3)

Supplementary Figure S2: Variation between co-culture spheroids in response to FFA treatment within one experiment - COL1A1

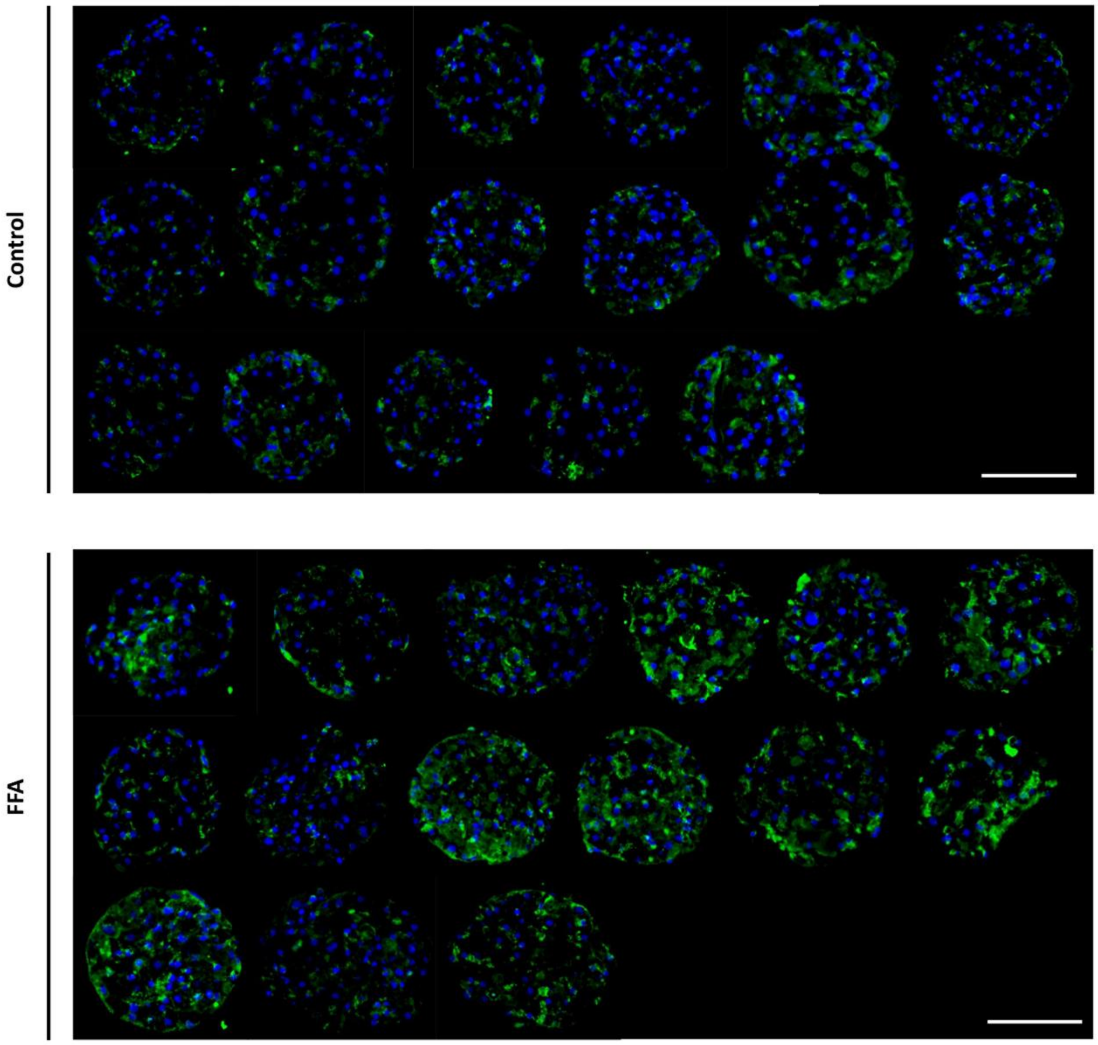

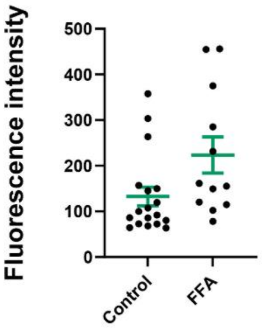

Supplementary Fig. S2: We show a great variability among spheroids in terms of response to chemical stimuli, in this case to FFA treatment with the consequential elevation in production of COL1A1 (lower panel) in comparison to the control spheroids (upper panel). Every image taken is analysed and quantified. Every dot in the plot on the left represents the quantified fluorescence intensity (integrated density of the respective staining divided by the area of the DAPI staining) of 1 stained slice of a spheroid in 1 experiment. The average of all quantified spheroids per condition per experiment is presented in the immunohistochemistry plots throughout the article. The size of the spheroids is marked with a white scale bar of 100 µm.

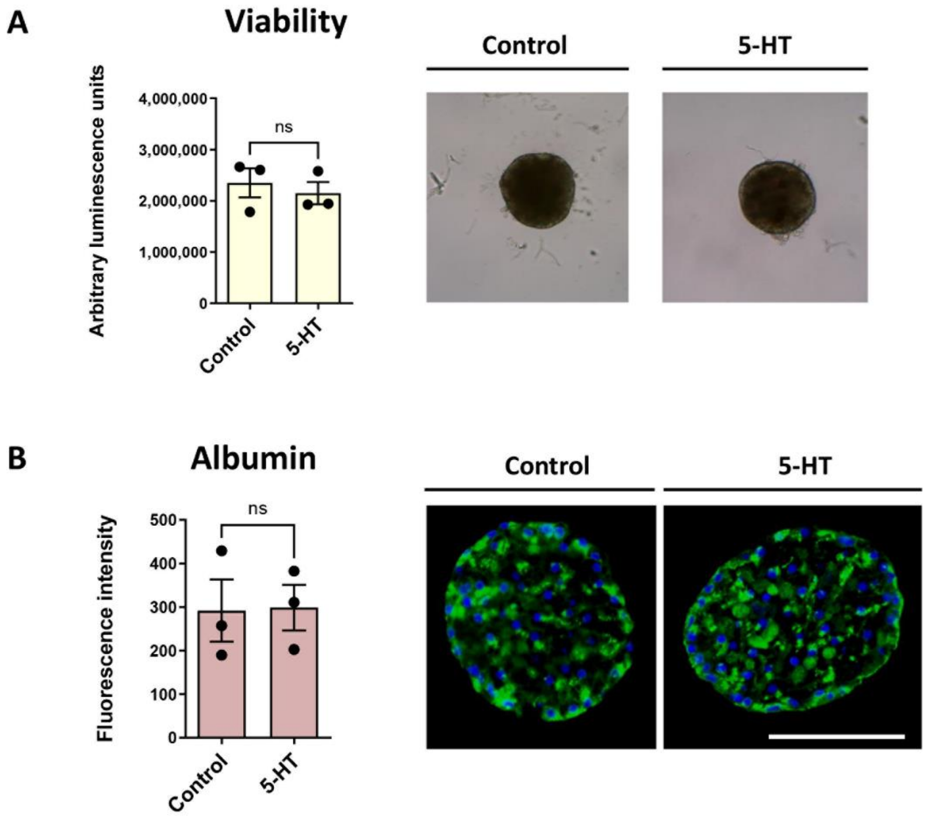

Supplementary Figure S3: Effect of 5-HT treatment on viability and functionality

(A) Treatment of spheroids with 5-HT for 7 days did not affect viability of spheroids, which was measured by quantification of the released ATP. One dot represents the average of the ATP measurements in at least 4 spheroids per condition per experiment. (n=3)

(B) Treatment of spheroids with 5-HT did not affect the functionality of hepatocytes in the spheroids, which was measured by the albumin staining. One dot represents the average of at least 10 imaged spheroids per condition per spheroid. (n=3)

Supplementary Table 1: TaqMan probes used for RT-PCR

Gene	TaqMan probe number	Company
COL1A1	Hs00164004_m1	Thermo Fisher
TGFB1	Hs00998133_m1	Thermo Fisher
VIM	Hs00185584_m1	Thermo Fisher
HTR2A	Hs06626790_s1	Thermo Fisher
LOX	Hs00942483_m1	Thermo Fisher
IL6	Hs00174131_m1	Thermo Fisher
TBP	Hs00427620_m1	Thermo Fisher

Supplementary Table 2: A list of antibodies used for immunohistochemistry

Primary Antibody	Species	Company	Cat. No.	Dilution
COL1A1	Rabbit	Abcam	ab34710	1:200
TGF-β1	Rabbit	Abcam	ab92486	1:200
α-SMA	Mouse	Abcam	ab7817	1:200
CTGF	Rabbit	Abcam	ab6992	1:300
Vimentin	Rabbit	Abcam	ab92547	1:200
Albumin	Mouse	Santa Cruz	sc-51515	1:200
5-HT_{2A}	Goat	Novus Biologicals	NBP2-26091	1:100

Secondary Antibody	Company	Cat. No.	Dilution
Alexa Fluor 488 Goat-anti-Rabbit	Thermo Fisher	A-11008	1:500
Alexa Fluor 555 Donkey-anti-Mouse	Thermo Fisher	A-31570	1:500
Alexa Fluor 488 Donkey-anti-Rabbit	Thermo Fisher	A-21206	1:500
Alexa Fluor 555 Donkey-anti-Goat	Thermo Fisher	A-21432	1:500

Supplementary Table 3: Serotonin quantification

			**5-HT concentration (nM)**
**Mono-cultures**	**Supernatant**	**Control**	0,4
		**FFA**	1,3
	**Lysate**	**Control**	0
		**FFA**	0
**Co-cultures**	**Supernatant**	**Control**	0
		**FFA**	1,2
	**Lysate**	**Control**	1,2
		**FFA**	0

Mono-cultures present spheroids comprised only of primary human hepatocytes, while co-cultures present spheroids comprised of both primary human hepatocytes and non-parenchymal cells in a designated ratio. Supernatants and lysates of spheroids were used as samples. Spheroids were either controls or treated with free fatty acids (FFA). Concentrations present an average of duplicate analyses.
